# Determining the Efficiency of Different Preoperative Difficult Intubation Tests on Patients Undergoing Caesarean Section

**DOI:** 10.4274/balkanmedj.2016.0877

**Published:** 2017-09-29

**Authors:** İlker Yıldırım, Mehmet Turan İnal, Dilek Memiş, F. Nesrin Turan

**Affiliations:** 1 Clinic of Anesthesiology, Uzunköprü Public Hospital, Edirne, Turkey; 2 Department of Anesthesiology and Reanimation, Trakya University School of Medicine, Edirne, Turkey; 3 Department of Biostatistics, Trakya University School of Medicine, Edirne, Turkey

**Keywords:** Caesarean section, difficult intubation, predictive tests

## Abstract

**Background:**

Pregnancy-induced anatomical and physiological changes in the airway make airway management difficult in obstetric patients; thus, preoperative evaluation of the airway is important for obstetric patients.

**Aims:**

To determine the effectiveness of the modified Mallampati test; the interincisor, sternomental and thyromental distances and the upper limb bite test. The second aim was to assess the effectiveness of the combination of the upper limb bite test with the other tests in obstetric patients.

**Study Design:**

Cross-sectional study.

**Methods:**

Pregnant women (n=250) scheduled for caesarean section were analysed. The patients’ ages, heights and weights were collected. Preoperative airway evaluation was done by using a modified version of the Mallampati test. The interincisor, sternomental and thyromental distances were measured, and the upper limb bite test was performed. The laryngoscopy difficulty was evaluated by using Cormack-Lehane classification.

**Results:**

No statistically significant differences were found between groups in age, height or weight (p>0.05). The modified Mallampati test and interincisor, sternomental and thyromental distances revealed a lower number of easy intubations than that determined by the Cormack-Lehane classification and a higher number of difficult intubations than the actual number of cases (p<0.05). The sensitivity and specificity of the modified Mallampati test, the upper limb bite test, the interincisor distance test and the sternomental and thyromental distance tests were found to be 73.08, 57.69, 84.62, 80.77 and 88.46 and 90.62, 99.11, 83.04, 84.37 and 87.05, respectively. When the combinations were examined, the sensitivity and specificity of the combination of the upper limb bite test with the modified Mallampati test were found to be 57.69 and 100, respectively. When the upper limb bite test was combined with the interincisor distance, the sensitivity and specificity were 46.15 and 100, respectively. We found a sensitivity and specificity of 93.75 and 95.30, respectively, for the combination of the upper limb bite test with the thyromental distance test. The sensitivity and specificity of the combination of the upper limb bite test with the modified Mallampati test and interincisor distance test were found to be 46.15 and 100, respectively. For combination of all the tests, the sensitivity and specificity was 42.31 and 100, respectively.

**Conclusion:**

When all combinations are evaluated in the decision of difficult intubation, the combination of the upper limb bite test and thyromental distance test is superior to the use of other methods alone to predict difficult intubation in pregnant women.

Difficult intubation is seen more often in obstetric patients than in other surgical patients and is the leading cause of anaesthesia-related maternal mortality. Pregnancy-induced anatomical and physiological changes in the airway make airway management in obstetric patients one of the important issues in anaesthesia practice. This condition arises from limited laryngoscopy movement due to oedema in the airways, a large tongue, fragile tissues and an enlarged abdomen. Failed intubation is very important in this group of patients in terms of increased morbidity and mortality. In the literature, considering pregnancy-related deaths, complications associated with anaesthesia practice are responsible for 2.5% of all maternal deaths, of which airway problems constitute 58%, of which endotracheal intubation failures constitute a major part (1,2,3). Therefore, the preoperative evaluation of the difficulty of intubation is important for patients undergoing caesarean section under general anaesthesia (4,5,6).

A variety of different tests are used to evaluate difficult intubation in advance. Among them are the Upper Lip Bite test (ULBT), the Modified Mallampati test (MMT), thyromental distance (TMD) measurement, sternomental distance (SMD) measurement and interincisor distance (IID) measurement. Several previous studies reported on the effectiveness and weaknesses of these tests (7,8,9,10,11,12). Different studies (8,9,10,11,12,13) concluded that the ULBT test is simple and easy to apply; therefore, we planned to combine the other tests with the ULBT test.

The first aim of the study was to determine the effectiveness of each of the single difficult intubation predictive tests, and the second aim was to determine the effectiveness of the combination of different predictive tests with the ULBT test in patients undergoing caesarean section under general anaesthesia.

## MATERIALS AND METHODS

The study was carried out with a total of two hundred-fifty pregnant women planned for caesarean section. Written informed consent was obtained between the dates 10.06.2011 and 01.09.2012. Ethical approval for this study (Trakya University ethical committe number: 2011/113) was provided on 08.06.2011.

Patients who had undergone neck or jaw surgery, burn or trauma related to the upper respiratory tract; had a condition leading to limited cervical mobility such as cervical disc herniation or rheumatoid arthritis; had ankylosing spondylitis or had a bulk in the pharynx, larynx or mouth were not included in the study.

The MMT, IID, SMD, TMD and ULBT tests were performed on the patients in the preoperative period and recorded by a research assistant (IY) who had at least 2 years of experience in anaesthesia. In addition, data on age, height and weight of the patients were collected.

The MMT as described by Samsoon and Young (7) classified the airway into four classes.

Class I: Soft palate, fauces, uvula and pillars visible, Class II: Soft palate, fauces and base of uvula visible, soft palate visible and hard palate visible. Classes I and II were predictive of easy intubation, and Classes III and IV were predictive of difficult intubation.

The IID was defined as the distance between the incisors when the patient's mouth was completely open, and less than 3-3.5 cm was regarded as a sign of a difficult intubation (8,9,10,11).

The SMD was defined as the distance between the middle point of the jaw and the upper limit of the manibrium sterni while the patient's head was in full extension, and the mouth was closed. Less than 12.5 cm was regarded as a sign of a difficult intubation (8,9,10,11).

The TMD was defined as the distance between the middle point of the tip of the jaw and the overhang of the thyroid cartilage while the patient's head was in full extension, and the mouth was closed; less than 6 cm was regarded as a difficult intubation (8,9,10,11).

The ULBT was described by Khan et al. (12) in 2003. In this test, Class 1: The lower incisors can bite the upper lip above the vermilion line. Class 2: The lower incisors can bite the upper lip below the vermilion line. Class 3: The lower incisors cannot bite the upper lip. Classes I and II were predictive of easy intubation, and Class III was predictive of a difficult intubation.

After pregnant women received general anaesthesia with 2 mg/kg propofol (Propofol; Fresenius Kabi medical, Hamburg, Germany) and 1 mg/kg succinylcholine (lysthenon; Fako Medical, İstanbul, Turkey) as in a standard in intubation, a rapid intubation was performed. Intubation was performed by one anaesthetist who was blinded to the study, using the Macintosh blade, and laryngoscopy difficulty was evaluated by the Cormack-Lehane classification, without applying cricoid pressure. Grade I: Full view of the glottis can be seen, Grade II: Partial view of the glottis can be seen, Grade III: Only the epiglottis can be seen and Grade IV: The epiglottis and glottis cannot be seen. Grades I and II were accepted as an easy airway, and Grades III and IV were accepted as a difficult airway. Patients were classified as easy intubations or difficult intubations using the Cormack grading system. Accordingly, those who had Cormack Grade I-II airways were classified as easy intubations, and those who had Cormack Grade III-IV airways were classified as difficult intubations (14).

Difficult intubation, as defined by the American Association of Anaesthetists, is three or more failed attempts during intubation or the process taking more than 10 minutes (15). Between failed intubation attempts, oxygen was given by mask ventilation to the patient. In the case of tracheal ventilation failure, mask ventilation or laryngeal mask airway was used for the patient. Bleeding, lacerations, dental trauma and airway trauma were all recorded.

The first aim was to determine the effectiveness of the single difficult intubation predictive tests and the second aim was to determine the effectiveness of the combination of different predictive tests with the ULBT test due to its simplicity and non-invasiveness. When both tests were positive in determining difficult intubation, the combined test was considered positive.

### Statistical analysis

Statistical evaluation was carried out using SPSS Statistics for Windows, Version 19.0. (IBM Corp., Armonk, NY: Released 2010). After the compliance of the data with the normal distribution was checked by a one-sample Kolmogorov-Smirnov test, a Mann-Whitney U test was used for non-normally distributed data. For the interincisor distance, sternomental distance and thyromental distance measurements, cut-off points were calculated by receiver operating curve analysis. The Kappa test for compliance between Cormack-Lehane classification and MMT, ULBT, IID, SMD and TMD was used, and the McNemar test was used for the difference between tests. Mean ± standard deviation were used, and results were considered significant at p<0.05. Post hoc evaluation indicated that a sample size of 248 subjects would achieve 87% power to detect a difference of -0.577 between two diagnostic tests with sensitivities of 0.423 and 1.000. This procedure uses a two-sided McNemar test with a significance level of 0.05. The prevalence of disease in the population is 0.104. The proportion of discordant pairs is 0.940.

## RESULTS

Two hundred-fifty female patients were included in the study. Patients were divided into two groups according to the Cormack-Lehane classification. A total of 224 (89.6%) patients were classified as easy intubations and 26 (10.4%) were classified as difficult intubations. All patients in the difficult intubation group were successfully ventilated, and in 4 of the 26 patients, blood was detected in the laryngoscope. No lacerations, dental trauma or airway trauma were recorded.

Mean patient age was 30.04±6.81 years in the easy intubation group and 30.42±6.92 years in the difficult intubation group. Mean patient height was 162.33±6.47 cm in the easy intubation group and 160.38±8.12 cm in the difficult intubation group. Mean patient weight was 81.00±17.24 kg in the easy intubation group and 82.12±20.44 kg in the difficult intubation group. When the groups were compared, no statistically significant difference was found between groups in age, height, or weight (p>0.05) (Table 1).

When the difficult intubation group was assessed by MMT, 203 (81.20%) of 224 patients were identified as easy intubations while 21 (8.40%) were identified as difficult intubations. Seven (2.80%) out of 26 patients were assessed as easy intubations and 19 of them (7.60%) were assessed as difficult intubations. According to IID measurements, 186 (74.40%) out of 224 patients were identified as easy intubations, and 38 (1.60%) were identified as difficult intubations. Four (1.60%) out of 26 patients were assessed as easy intubations, and 22 (8.80%) were assessed as difficult intubations. We found the cut-off point for the IID test to be 4.5 cm. The AUC value was 0.926. When SMD was used, 189 (75.60%) out of 224 patients were identified as easy intubations, and 35 (14.00%) were identified as difficult intubations. Five (2.0%) out of 26 patients were assessed as easy intubations, and the other 21 (8.4%) were assessed as difficult intubations. In our study, the cut-off point for the SMD test was 13.5 cm. The AUC value for SMD was 0.888. According to TMD measurements, 195 (78.0%) out of 224 patients were identified as easy intubations and 29 (11.6%) were identified as difficult intubations. Three (1.2%) out of 26 patients who were classified as difficult intubations according to the Cormack-Lehane classification were identified as easy intubations, and 23 of them (9.2%) were assessed as difficult intubations. The cut-off point for the TMD was 6.5 cm. The AUC was 0.901. When the difficult intubation group was assessed by ULBT, 222 (88.80%) out of 224 patients were identified as easy intubations, and 2 (0.80%) of them were identified as difficult intubations. Eleven (4.40%) patients were assessed as easy intubations, and 15 (6.00%) were assessed as difficult intubations. When the groups were compared statistically, the MMT, IID, SMD and TMD revealed a lower number than the case number of easy intubations and a higher number than the case number of difficult intubations as determined by the Cormack-Lehane classification. The ULBT revealed a higher number than the case number of easy intubations and a lower number than the case number of difficult intubations (p<0.05) (Table 2). The sensitivity, specificity and positive and negative predictive values are shown in Table 3.

The relationships of all intubation tests with difficult intubations by having two or more combinations with the ULBT were investigated statistically. When the ULBT and MMT were combined, 11 cases of easy intubation were identified as difficult intubations, while 15 cases were found to have the probability of difficult intubation with the binary test. The difference between the possible cases of difficult intubation and the actual number of difficult intubations was found to be statistically significant (p=0.001, Table 4) The sensitivity, specificity, PPV and NPV were 57.69%, 100%, 100% and 95.32%, respectively (Table 5). If ULBT and IID were combined, in the binary test, 14 cases of easy intubation were identified as difficult intubations, and 12 cases were identified as probable difficult intubations. A statistically significant difference was detected between possible cases of difficult intubation and the actual number of difficult intubations (p<0.001, Table 4). We found the sensitivity, specificity, PPV and NPV to be 46.15%, 100%, 100% and 94.12%, respectively (Table 5). When ULBT and SMD were combined, 12 cases of easy intubation were identified as difficult intubations, while 15 cases were identified as probable difficult intubations. The difference between the possible cases of difficult intubation and the actual number of difficult intubations was statistically significant (p=0.003, Table 4). The sensitivity was 53.85%, specificity was 99.55%, PPV was 93.33 and NPV was 94.89% (Table 5). If ULBT and TMD were combined, 11 cases of easy intubation were identified as difficult intubations, while 16 cases were identified as probable difficult intubations. A statistically significant difference was detected between the possible cases of difficult intubation and the actual number of difficult intubations (p=0.006, Table 4). The sensitivity, specificity, PPV and NPV were 93.75%, 95.30%, 57.69 and 99.55%, respectively (Table 5).

In the statistical comparisons using Cormack grade performed with the ULBT, MMT and IID, in the ternary test, 14 cases of easy intubation were identified as difficult intubations, while 12 cases were identified as probable difficult intubations. The difference between the possible cases of difficult intubation and the actual number of difficult intubations was statistically significant (p<0.001, Table 4). The sensitivity was 46.15%, the specificity was 100.00%, the PPV was 100.00% and the NPV was 94.12% (Table 5). When ULBT was combined with the MMT, IID and SMD, in the quaternary test, 15 cases of easy intubation were identified as difficult intubations, while 12 cases were identified as probable difficult intubations (Table 4). A statistically significant difference was detected between possible cases of difficult intubation and the actual number of difficult intubations (p<0.001; sensitivity: 43.31%; specificity: 100.00%; PPV: 100.00; NPV: 93.72%; Table 5). If the five tests were combined, 15 cases of easy intubation were identified as difficult intubations, while 12 cases were identified as probable difficult intubations (Table 4). The difference between the possible cases of difficult intubation and the actual number of difficult intubations was statistically significant (p<0.001; sensitivity: 43.31%; specificity: 100.00%; PPV: 100.00%; NPV: 93.72%; Table 5).

There was moderate agreement between the Cormack-Lehane classification and the ULBT, MMT, IID, SMD combination and the ULBT, MMT, IID, SMD, TMD combination, while there was high agreement between the Cormack-Lehane classification and other assessments (Table 4).

## DISCUSSION

Unanticipated difficult endotracheal intubation in the anaesthesia practice is an undesirable and life-threatening condition. The most common cause of anaesthesia-related morbidity and mortality is airway inability after induction (15).

Shiga et al. (5) identified difficult intubation cases at a frequency of 5.8% in a meta-analysis comprising 50.760 patients. Iohom et al. (6) identified difficult intubation in 9% of 212 patients. Mallampati et al. (16) predicted difficult intubation in 28 out of 210 cases in direct laryngoscopy, a rate of 13.3%. In our study, the incidence of difficult intubation was 10.4%. The main reason for this situation may be the increase in the incidence of a physiologically difficult airway due to airway oedema in pregnant women. Another reason is that the preanaesthetic evaluation of all the cases was not performed by the same person.

Whether laryngoscopy will be easy or difficult may be predicted by using the Mallampati classification (16). Although the Mallampati test is not considered as reliable as in the past, due to interobserver variability, the patient's position, mounding of the tongue during the procedure or neck mobility, it is still a convenient and practical method that is easily applied at the bedside. Merah et al. (4) performed five bedside monitoring tests in 80 obstetric patients and found 87.1% sensitivity and 99.6% specificity of the Mallampati test. They concluded that the Mallampati test could be used to predict difficult intubation. Frerk (17) stated that MMT was not specific for routine use, because it had high sensitivity but produced many false-positive (1/5) results with 81.2% sensitivity and 81.5% selectivity. In our study, we found the MMT to have 73.08% sensitivity, 90.62% specificity, 47.50% PPV and 96.67% NPV. Possible explanations for the disparate results are the number of cases, failure to establish good communication with patients, different patient groups and interobserver variability.

The ULBT is a new, simple and easily applied test. Eberhart et al. (18) found 28.2% sensitivity, 92.5% specificity, 33.6% PPV and 90.6% NPV in their study. Hester et al. (13) found 55% sensitivity, 97% specificity and 83% PPV and stated that the test was applicable because it was easy to apply. For the ULBT, Salimi et al. (19) found 70% sensitivity, 93% specificity, 39% PPV and 98% NPV in their study. We found 57.69% sensitivity, 99.11% specificity, 88.24% PPV and 95.28% NPV for the ULBT. In a recent study conducted by Khan et al. (20) they found 78.91% sensitivity, 91.96% specificity, 91.05% PPV and 98.8% NPV for ULBT. The investigators stated that the ULBT alone was not sufficient to predict difficult intubation with these results but would be useful for distinguishing easy intubation cases because it had high sensitivity and negative cut-off values. The practicality of the ULBT and the correlation of its results with those of the MMT demonstrate its usefulness in predicting difficult intubation in pregnant women. Moreover, Allahyary et al. (11) found that the ULBT as a single test was highly sensitive (94.6%) and specific (97.6%), and as a result, the ULBT was reported to be a valuable marker for predicting difficult laryngoscopy.

Yıldız et al. (10) found a significant difference between the IID values of the difficult and easy laryngoscopy cases when they confirmed the cut-off value as 45 mm for IID. Wilson et al. (21) found an increased risk of difficult laryngoscopy in patients with IID smaller than 5 cm. Asık et al. (22) found that patients who had IID values less than 30 mm had statistically significantly higher cut-off values than those with higher IID values. This study reported a sensitivity of 36%, a specificity of 81%, PPV of 35% and NPV of 81%. For the IID measurement for predicting difficult intubation in pregnant women, we found 84.62% sensitivity, 83.04% specificity, 36.67% PPV and 97.89% NPV. We propose that lower threshold values and the different numbers of cases in other studies explain the disparate results between the studies.

The extension of the head is an important parameter in predicting whether the intubation will be easy or difficult. SMD may be an indicator of head and neck mobility (23). Shiga et al. (5) found a moderate level of sensitivity for SMD (62%), 82% specificity, higher PPV and lower NPV compared with the other tests and argued that it was the best test for predicting difficult intubation. Al Ramadhani et al. (23) evaluated the Cormack grade classification and SMD of 523 patients who underwent emergency or elective caesarean section and considered an SMD of 13.5 cm or less as the threshold for predicting difficult intubation. The authors also reported a sensitivity of 66.7%, a specificity of 71.1%, PPV of 7.6% and NPV of 98.4%. Savva (24) reported that SMD should be used as the single objective indicator of difficult intubation. This study reported a sensitivity of 82.4%, a specificity of 88.6% and a PPV of 26.9%, provided that SMD was less than 12.5 cm. In our study, the cut-off value for SMD was 13.5 cm, the sensitivity was 80.77%, the specificity was 84.37%, PPV was 37.50% and NPV was 97.42%. Therefore, we believe that the disparate results may have been due to the use of different cut-off values and the failure to bring the patient's head into sufficient extension.

Yıldız et al. (10) reported that the probability of a difficult intubation is high when TMD is between 6 and 6.5 cm and impossible when it is below 6 cm. Frerk (17) mentions the probability of a difficult intubation when TMD is under 7 cm. We identified the cut-off value for TMD as 6.5 cm. Tse et al. (25) reported that TMD ≤7 cm was not a good predictor of difficult intubation preoperatively, as it had a low sensitivity (32%), PPV of 20%, high specificity (80%) and NPV of 89%.

Wong and Hung (26) conducted a study to assess the prevalence and prediction of difficult intubation in Chinese pregnant and non-pregnant patients. They found no difference in the rates of difficult intubation and concluded combination of predictive tests for assessing difficult intubation. They used the IID and TMD in their study and found a statistically significant difference in IID values. The authors also found sensitivity and specificity as high as 71% and 92%, respectively, when TMD <5.5 cm was taken, whereas PPV was only found to be 11%. We found that the sensitivity for TMD was 88.46%, the specificity was 87.05%, PPV was 44.23% and NPV was 98.48%.

Since a single test is not sufficient to predict difficult intubation, it was reported that the most accurate results would be obtained by a combination of tests (5). In the study by Iohom et al. (6), when used alone, the MMT, TMD and SMD tests were weak in terms of sensitivity, specificity and PPV. When MMT was combined with TMD or SMD, the sensitivity decreased, but the negative cut-off value remained at 93%. They found that the specificity and positive cut-off value for the MMT with the TMD increased from 89% to 100% and increased from 27% to 100% with SMD. They reported that, when TMD and SMD were used together, the sensitivity decreased (33%), the specificity increased (98%) and PPV increased (67%). They concluded that the MMT, TMD and SMD tests would be useful routine preoperative tests in predicting difficult intubation. Yıldız et al. (10) found that the combination of MMT and TMD had a high positive cut-off value (50%), as the combination of MMT, IID and TMD had the highest positive cut-off value among all combinations (66.7%). Frerk (17) concluded that the combination having the highest sensitivity and selectivity was the MMT and TMD measurement. in conjunction with the MMT, the sensitivity was 57.69%, the specificity was 100.00%, PPV was 100.00% and NPV was 95.32%. When TMD and the ULBT were used together, the sensitivity was 93.75%, specificity was 95.30%, PPV was 57.69% and NPV was 99.55%.

When all combinations are evaluated in the decision of difficult intubation, the combination of the ULBT and TMD methods is more suitable to use than other methods alone to predict difficult intubation in pregnant women.

## Figures and Tables

**Table 1:**
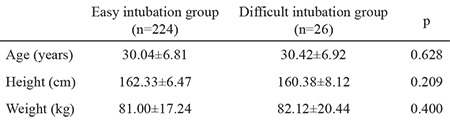
Demographic data

**Table 2:**
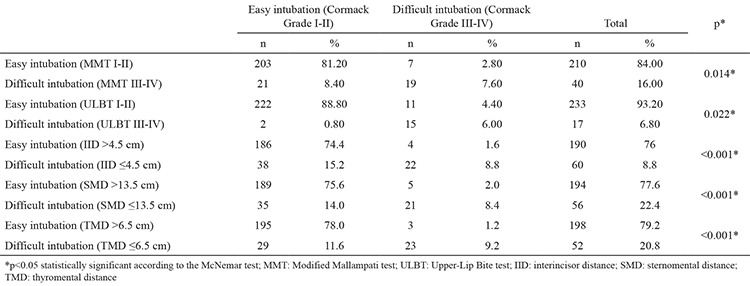
Comparison of the groups according to the MMT, ULBT, IID, SMD and TMD

**Table 3:**
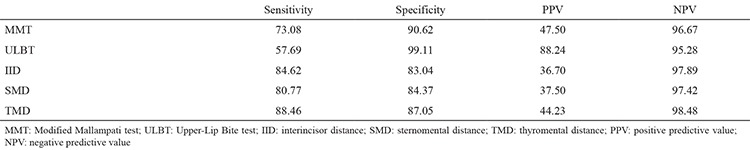
Comparison of sensitivity, specificity and positive and negative cut-off values of all groups

**Table 4:**
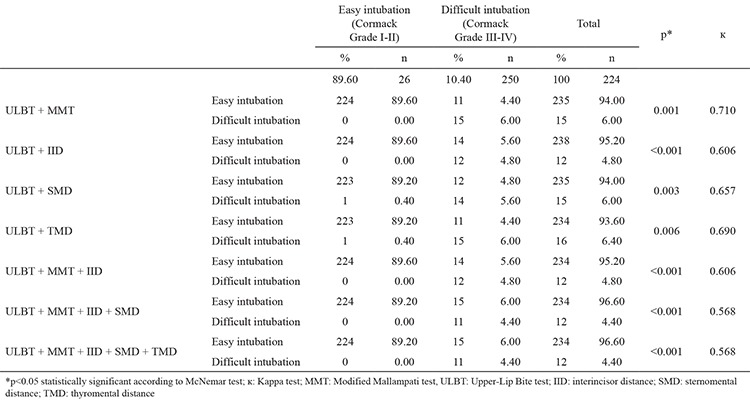
Comparison of the group combinations

**Table 5:**
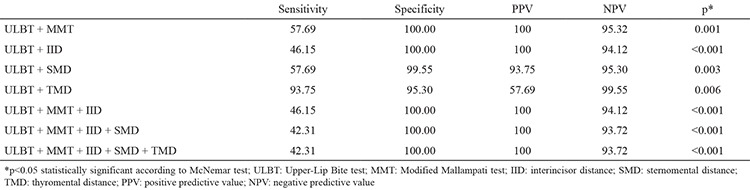
Comparison of group combinations according to sensitivity, specificity and positive and negative predictive values
